# Harnessing the Helminth Secretome for Therapeutic Immunomodulators

**DOI:** 10.1155/2014/964350

**Published:** 2014-07-15

**Authors:** Dana Ditgen, Emmanuela M. Anandarajah, Kamila A. Meissner, Norbert Brattig, Carsten Wrenger, Eva Liebau

**Affiliations:** ^1^Department of Molecular Physiology, Westfälische Wilhelms-University Münster, Schlossplatz 8, 48143 Münster, Germany; ^2^Unit for Drug Discovery, Department of Parasitology, Institute of Biomedical Science, University of São Paulo, 1374 Prof. Lineu Prestes Avenue, 05508-000 São Paulo, SP, Brazil; ^3^Bernhard-Nocht-Institute, Bernhard-Nocht-Straße 74, 20259 Hamburg, Germany

## Abstract

Helminths are the largest and most complex pathogens to invade and live within the human body. Since they are not able to outpace the immune system by rapid antigen variation or faster cell division or retreat into protective niches not accessible to immune effector mechanisms, their long-term survival depends on influencing and regulating the immune responses away from the mode of action most damaging to them. Immunologists have focused on the excretory and secretory products that are released by the helminths, since they can change the host environment by modulating the immune system. Here we give a brief overview of the helminth-associated immune response and the currently available helminth secretome data. We introduce some major secretome-derived immunomodulatory molecules and describe their potential mode of action. Finally, the applicability of helminth-derived therapeutic proteins in the treatment of allergic and autoimmune inflammatory disease is discussed.

## 1. Introduction

During the last centuries living conditions in western countries changed extremely and social and economical structures shifted dramatically. As a suggested consequence of the resulting improvements in hygiene, antiparasite treatments, and the reduced exposure to pathogens and childhood infections, the occurrence of chronical inflammatory diseases and allergies increased rapidly [1, 2]. In 1989, David Strachan was the first one to link these two developments and enunciated the “*Hygiene Hypothesis.*” According to this thesis, the observed increases in certain inflammatory disorders were due to the decreased early-life exposure to microorganisms and other eukaryotic infectious agents including helminths [3].

Worm-like parasites that belong to unrelated phyla, namely, the plathelminthes (trematodes and cestodes) and the nematodes, were already present in early Hominidae. This long coexistence between humans and helminths must have had a fundamental impact on the constitution and regulation of the immune system [4–6].

As an advancement of the “*Hygiene Hypothesis,*” the “*Old Friend Hypothesis*” was put forward by Graham Rook. He hypothesized that numerous harmless pseudocommensals, including the helminths, were tolerated by the immune system due to their abundant presence [6]. In this way, the tolerance of helminths reduces the negative impact on the host's fitness, since it decreases the tissue damage or other fitness costs [8].

Recently, William Parker extended this hypothesis to the “*Lost Friends Theory*” or the “*Biome Depletion Theory.*” This theory describes the consequences of separating us from our partners in coevolution. Accordingly, the reduced pattern of exposure to microorganisms and helminths and their depletion from the human ecosystem lead to an unstable and unbalanced immune state [9]. Since the loss of components of our biome is partly responsible for epidemics of immune-related diseases such as autoimmune and allergic diseases, the most reasonable solution would be the restoration of the biome [10]. Hence exposure to helminth parasites could again establish and maintain the normal immunological balance in humans. However, colonization with intestinal helminths as immune therapy is problematic due to various physiological side effects. Furthermore, the induced immune hyporesponsiveness could affect immune reactions to concomitant infections and vaccination efficacies [4, 11]. An alternative approach therefore is to identify the immune modulatory molecules produced by helminths that can alter immune functions.

## 2. Helminths

Infections with helminth parasites have great impact on global health and it has been estimated that at least one-third of the human population is infected with these parasites, prompting helminth infections to be termed the “*Great Neglected Tropical Diseases*” [4, 12]. Although highly parasitized individuals can suffer from severe pathology, helminths usually cause asymptomatic or subclinical chronic infections, with little evidence of an inflammatory response or overt tissue destruction. As such, many helminths can survive within their host for decades.

About one-third of mankind in the tropics and subtropics are chronically infected with one or more helminths [4, 12]. According to the WHO, more than 1.5 billion people or 24% of the world's population are infected with soil-transmitted infections (WHO, report 2014). The most common helminthiases of humans are caused by soil-transmitted nematodes, namely,* Ascaris lumbricoides*,* Trichuris trichiura*, and the hookworms* Necator americanus* and* Ancylostoma duodenale*, followed by schistosomiasis (blood flukes of the genus* Schistosoma*) and lymphatic filariasis (*Wuchereria bancrofti*,* Brugia malayi*, and* Brugia timori*) [13] (Table 1). According to the CDC, approximately 807–1,121 million people are infected with* A. lumbricoides*, 604–795 millions with whipworms, and 576–740 millions with hookworms (CDC, report 2013).

While these helminths show a remarkable variety in their mode of life, their hosts, and life history stages, they induce a canonical host immune response pattern.

## 3. Helminth-Associated Immune Response

The human immune system responds to the invasion of helminths into the organism differently than to bacterial or viral infections. While microbial pathogens are usually eliminated from the host with a rapid and inflammatory immune response, the immune response to helminths is less severe and has a strong regulatory character [14].

Worm infections elicit T_H_2 cell responses associated with a significant production of IL-4, IL-5, IL-9, IL-13, IL-31, IL-25, and IL-10 [13, 15]. Furthermore, the worm infections are often associated with high levels of IgE, IgG1, and IgG4 and stable eosinophil and mast cell responses [16]. Eosinophils become activated in helminth-infected sites and secrete proinflammatory cationic proteins, oxygen radicals, lipids, and other mediators like cytokines. Eosinophils and mast cells release their cytotoxic products during degranulations at infected sites [17]. The release of mediators leads to blood vessel enlargement, increased mucus production, and cell contraction of smooth muscle cells [18]. It is assumed that the primary role of eosinophils lies in the defence against large organisms which cannot be phagocytosed. Eosinophils can bind to carbohydrate ligands and fixed antibodies on the parasites surface, degranulate and release their cytotoxic agents to harm the parasite [19], and then get phagocytosed by macrophages after their response [17, 18].

Within 24 h after penetration into the host organism most helminths trigger an immediate production of T_H_2 cytokines [14]. The protective effect of helminths against allergy and autoimmunity strongly depends on worm species (age, state of infection, and parasite burden) [20, 21]. Individuals infected with filarial nematodes like* W. bancrofti* and* Onchocerca volvulus* or with trematodes like* Schistosoma mansoni* and* Schistosoma japonicum* develop a strong T_H_2 immune response [22]. Nevertheless, three helminth stages are known, which do not induce a T_H_2 response immediately after infection: the cercariae of schistosomes, the microfilarial stage of* B. malayi,* and the nematode* Trichuris muris* [14].

In case of helminth and* Mycobacterium tuberculosis* coinfection, a dramatic reduction of protective immune responses can be observed [22]. However, some infections with parasitic worms like* Nippostrongylus brasiliensis* and* Toxocara canis* with* Mycobacterium bovis* or* M. tuberculosis* do not lead to an impaired protective immune response [22–24].

Although allergy-associated T_H_2 responses and antihelminthic T_H_2 responses are very similar, they also differ as follows: (1) larger amounts of polyclonal, non-parasite-specific IgE antibodies are produced that do not cause allergic reactions and (2) during helminth infection an induction of strong inflammatory regulatory immune responses occurs [25, 26]. In worm infections the Fc*ε* receptors on mast cells can be saturated with non-worm-specific IgE; thereby, a binding of worm-specific IgE is averted. This occupation of receptor-binding sites suppresses the immediate hypersensitivity responses and the degranulation of mast cells (IgE blocking hypothesis) [18]. The IgE blocking hypothesis is still a matter of discussion. Larson and colleagues have shown that in mice the suppression of basophil responsiveness by chronic helminth infections was found to be dependent on host IL-10 [27]. IL-10 downregulates key-IgE signaling molecules [27] causing the level of serum IgE to decrease. This in turn influences the production of IgE receptors on basophils and mast cells [28–30]. Additionally, Mitre and coworkers demonstrated that the blocking of FcER1 on mast cells and basophils by parasite-induced polyclonal IgE does not mediate the protection against atopy, since the ratio of polyclonal IgE to allergen-specific IgE is too low to saturate the receptors and to suppress degranulation of mast cells and basophils [28].

Furthermore, Larson and colleagues compared the release of histamine from basophils in helminth-infected children before and after anthelminthic drug treatment and observed the suppression of basophil responsiveness during the intestinal helminth infection. They proposed that this inhibition of basophils, which are involved in the development of T_H_2 responses and function as effector cells for allergy, leads to protection against allergic diseases [31].

Helminth parasites have developed a lot of strategies to evade or modulate the host immune responses with advantages on both sides [32]. Thus, there is a shift in the T_H_2 response towards immunosuppression, immunological tolerance, or modified T_H_2 response [16]. In case of immunosuppression an upregulation of regulatory T cells takes place which suppresses protective T_H_2 as well as inflammatory T_H_1 responses. During immunological tolerance development, effector T_H_2 cells enter a state of anergy and fail to develop specific T effector cells which mediate resistance. Finally, in the modified T_H_2 response, downstream effects of the normal T_H_2 responses are muted and result in an increase of noncomplement fixing IgG4 and IL-10 [16, 33, 34]. In case of asymptomatic parasitic infections, the concentration of the T_H_2-dependent isotype IgG4 is increased. A differential stimulation of IgG4 is promoted by IL-10 which is formed at high concentrations during chronic helminth infections [18]. Furthermore, many studies have shown that these helminth-mediated T_H_2 responses can also prevent the often harmful inflammatory T_H_1 responses by inducing suppressive regulatory T cells which contribute to the formation of IL-10 and TGF-*β*. Thus, helminths are able to regulate the immune responses and ensure homeostasis under various disease conditions such as autoimmune diseases, inflammations, cancer, and microbial infections [13, 15, 35].

Affected by IL-4, IL-13, and IL-21, the differentiation of alternative activated macrophages (AAMs) occurs that can inhibit the proliferation of other cells and support an increased intracellular growth of bacteria [13]. In addition to their recruitment to sites of infection and various effector functions, they also have strong anti-inflammatory properties. These are manifested by the secretion of IL-10 and TGF-*β* and the expression of certain genes that are involved in the repair of the extracellular matrix, fibrosis, and wound healing [13, 15]. Thus, AAMs serve tissue homeostasis, act as effector cells against parasites, and downregulate the adaptive immune system [16].

In summary, chronic helminth infections result in a downregulation of proinflammatory responses, an enhanced T_H_2 response, and repair mechanisms [13, 32].

**Figure 1:**
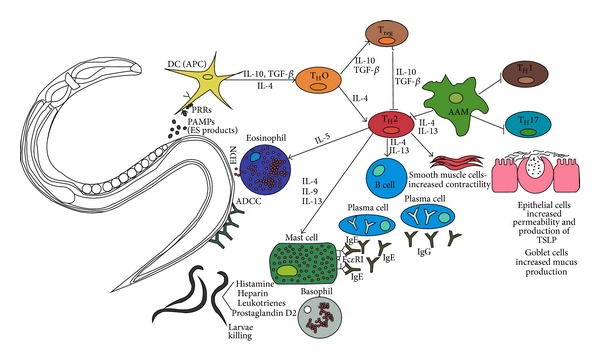
Cellular interactions in the immune response against helminths. Helminth-secreted excretory/secretory (ES) products are capable of inhibiting* in vitro* generated dendritic cells (DCs). They can inhibit the maturation of DCs and induce the expansion of functional T_regs_ [35, 36]. The helminth-induced T_H_2 response starts with the recognition of parasitic pathogen-associated molecular patterns (PAMPs) by certain pattern recognition receptors (PRRs) that are expressed on the DCs of the host [13, 37]. Through contact with the antigen, the DCs become activated, allowing them to act as antigen-presenting cells (APC) after the migration to the adjacent lymph nodes, with the ability of processing and presenting the antigen to T cells to initiate an immune response [16]. The helminth-induced host immune response is focused on the protection of the host organism and is mediated by T_H_2 cells. This response includes IL-4, IL-5, IL-13, and IL-10 secretion and production of IgG4 and IgE by B cells, as well as the activation of effector cells such as mast cells, eosinophils, and basophils [35]. Affected by IL-4 and IL-13 occurs the differentiation of alternatively activated macrophages (AAMs) which can inhibit the proliferation of other cells like T_H_1, T_H_2, and T_H_17 cells. Thus, these cells have strong anti-inflammatory properties, which are manifested by the secretion of IL-10 and TGF-*β* as well as the expression of additional genes [13, 16, 32]. Furthermore, IL-4 and IL-13 lead to an increased contractility of smooth muscle cells and a hypersecretion of mucus for expulsion of intestinal helminths [38]. Immune complexes of IgE bind to high affinity IgE receptors (Fc*ε*RI) on mast cells and basophils; this leads to an activation of these cells and a secretion of inflammatory mediators like histamine, heparin, leukotrienes, and prostaglandin D2 [16, 38–40]. PAMPs: pathogen-associated molecular patterns; PRRs: pattern recognition receptors; ES: excretory/secretory; IL: interleukin; Ig: immunoglobulin; AAM alternatively activated macrophages; T_H_: T helper cells; TGF-*β*: transforming growth factor-*β*; ADCC: antibody dependent cellular cytotoxicity; EDN: eosinophil derived neurotoxin; DC: dendritic cell; APC: antigen-presenting cell; T_reg_: regulatory T cell.

Figure 1 describes the interactions in the immune response to helminths.

## 4. Therapeutical Use of Helminths

Since there was such mounting evidence that helminth infections can modulate the mammalian immune response, treatment of immune dysregulatory diseases with live worms was considered to possess therapeutic capability, even though the suppression of an ongoing dysregulated immune response is probably more difficult to achieve than the prevention of its development. Because of the predicted lack of pathogenicity of certain helminth species, these were used in a series of clinical trials. For ethical reasons only individuals were treated who already suffered from immune dysregulatory diseases and in most studies the helminth dose was much lower than in natural infection [41, 42].

In the beginning, in a small trial three patients suffering from ulcerative colitis were treated with ova from the pig whipworm* Trichuris suis* [43]. In a clinical trial carried out by Summers et al.,* T. suis* ova (TSO) were administered to 29 patients suffering from Crohn's disease. 79.3% improved significantly and 72.4% experienced remission [44, 45]. Similar results were obtained in a larger trial where patients with ulcerative colitis were treated. A decrease of pathological symptoms was observed among 43.3% of the 54 patients treated with TSO [46]. Further double-blinded placebo-controlled clinical trials using TSO are currently conducted by Coronado Biosciences and Falk Pharmaceutical company [47] (http://www.clinicaltrials.gov). A different approach, using 50 live* N. americanus* larvae, was executed by Croese and colleagues with 9 patients suffering from Crohn's disease. Following the treatment, a decrease in pathology was recorded for two patients [48]. Correale and Farez conducted studies with multiple sclerosis patients that had also been affected by parasites. They were able to show that in these patients the disease pattern was weaker than in the control group [49, 50].

Nacher et al. observed that malaria patients with an additional gastrointestinal helminth infection, notably* Ascaris*, rarely showed acute renal failure or cerebral malaria in comparison to other malaria patients [51]. In mice infected with* Helicobacter pylori*, helminth infections were shown to reduce the tissue-damaging inflammation [52]. Recent epidemiological studies have clearly demonstrated that helminth, for example,* Schistosoma* spp., infected children had a reduced prevalence of allergic disorders. Other studies have shown that chronic infections with helminths protect people against allergic sensitization. The same results were achieved by infecting mice with* Strongyloides stercoralis* [25, 26]. Here, anthelmintic treatment led to loss of immune suppression and to an increase in atopic reactivity to allergens. Furthermore, the relationship between suppression of allergies and* Schistosoma* infection has been shown in both infected humans and mouse models [53].

A suppression of lung inflammation was shown in* S. stercoralis*-infected mice [54]. Also, extracts of the porcine parasite* Ascaris suum* inhibit IgE antibody production against unrelated antigens or antigens without reference and the generation of ovalbumin-specific T_H_2 responses in a murine model of asthma [25, 55]. Infection with the rodent intestinal nematode* N. brasiliensis* is another example of suppression of T_H_2 type allergic reactions, which inhibits the development of allergen-induced airway eosinophilia [56]. ES products of* N. brasiliensis* (NES) elicit a T_H_2 response by affecting DCs. But besides the regulation of T_H_2 response, NES also affect the proinflammatory T_H_1 responses by suppressing mitogen-dependent IFN-*γ* release as well as DCs produced and LPS induced IL-12p70 [57–59].

The trematode* Fasciola hepatica* causes liver fluke disease in sheep and cattle.* F. hepatica* infected mice, which were experimentally coinfected with* Bordetella pertussis,* showed a reduced bacterial-specific T_H_1 response. Furthermore, the mice were unabled to eliminate the microbe [60, 61]. This might be triggered by* F. hepatica* tegumental antigens that inhibit mast cells [62]. Contrariwise,* F. hepatica* did not suppress the IFN-*γ*-driven T_H_1 response triggered by* Toxoplasma gondii* infection [63].

As described before, helminths can downregulate harmful T_H_1 responses which are upregulated during autoimmune diseases. A therapeutic use of helminths could lead to a modified T_H_2 response and to an induction of T_regs_. This could result in a simultaneous reduction of T_H_1/T_H_17 responses and thereby reduce the pathology of autoimmune diseases [64–66].

In summary, all these studies support the concept of bystander immunoregulation by chronic helminthic infections being able to control allergen-specific or other inflammatory responses [67]. Since the dampening of the systemic immune response of the host is beneficial in transplantation, recent publications even suggest the use of helminthic therapy or helminth product therapy to enhance the allograft tolerance [68]. Despite these promising trials, the use of helminths within the therapeutical range is currently not possible due to various reasons: the breeding of helminths in the required amounts is not feasible and there are safety factors that need to be considered. Since there is evidence that only chronic but not acute infections are protective, parasite loss over time needs to be monitored [37]. The parasitic modes of action within the host are hardly explored and in some cases even completely unknown, so that possible side effects like diarrhea and intestinal pain are unpredictable [41, 69]. Unfortunately, most of the current experiments were performed with animal models and the assignability on humans cannot be guaranteed [70]. Furthermore, the psychological burden of the patients needs to be considered here as well [11, 25, 26].

The most potent anti-inflammatory response observed in humans is caused by chronic helminth infections, such as with* Schistosoma* spp. or* O. volvulus* and not by a transient infection. Therefore, it is obvious that only chronic infections with long-living helminths offer great therapeutic and preventive antiallergic effects [25, 26]. But not only live parasites can modulate or suppress the immune response. Glycans of the cuticula as well as helminth eggs or soluble extracts of worms can have the same effect. For example,* S. mansoni* egg soluble antigen (SEA) has the ability to prevent autoimmune type 1 diabetes by inducing a stronger T_H_2 and T_reg_ cell response as well as functional changes in APCs [65, 71–73]. However, the repeated use of helminth antigens might also induce neutralizing antibodies, thereby preventing long-term protection. In order to avoid the possibly critical therpeutic infection with a parasite, one major research aim is to identify and characterize helminth-derived molecules that are capable of modulating the immune system and to implement therapeutic approaches based on such molecules and thus replicate the protective effect already observed in helminth therapy. These immunomodulators could lead to the generation of novel strategies for anti-inflammatory drug development [41, 58, 70, 74, 75].

## 5. Excretory/Secretory (ES) Products

The immunomodulatory potency of helminths appears to be largely achieved by their surface or ES products [25]. Secretory products are substances with certain biological functions that are secreted from cells or glands. Contrariwise, excretory products are unnecessary metabolic products that are released from the body. Both, however, are sometimes difficult to distinguish from one another. The composition of these products varies significantly from parasite to parasite, but in general all of them contain different glycoproteins, proteins, and smaller peptides; nonprotein components include glycans, glycolipids, and bioactive lipids, like the eicosanoid inflammatory mediators, prostaglandins, and leukotrienes [76, 77]. The term ES products describes both substances that are actively secreted by helminths and products that are released within the course of physiological processes, for example, digestion or egg-laying [58, 78]. Furthermore, varying compositions of ES products at different life cycle stages can be expected [78, 79].

Given below are a few examples of ES products that exert the antiallergic and anti-inflammatory effects of helminth infections. In a chemically induced colitis mouse model the ES products of the canine hookworm* Ancylostoma caninum* reduced the inflammatory response and expression of proinflammatory cytokines while inducing the production of IL-4 and IL-10 [32, 75]. Furthermore, the ES products of the hookworm* Ancylostoma ceylanicum* can protect against chemically induced colitis by downregulating T_H_1 and T_H_17 cytokines [80]. Similar protection against inflammation was also obtained by using recombinant ES protein rTsP53 from* T. spiralis* in a colitis model [81]. Hsieh and associates also describe a secretory protein from* N. americanus* which binds to natural killer cells and stimulates the production of interferon-gamma [82]. The secreted protease inhibitor cystatin from* Acanthocheilonema viteae*, Av17, modulates macrophage-mediated inflammation in a murine model of colitis and significantly reduces inflammatory infiltrations and epithelial damage. As immunomodulatory strategy, the enhancement of IL-10 production by macrophages is proposed [83]. The immunomodulatory effect of ES products has also been shown for the cestode* Taenia crassiceps*.* T. crassiceps* ES products regulate DC activity by binding multiple receptors (e.g., MGL, MR, and TLR2), thereby downregulating TLR-mediated DC maturation and secretion of IL-12 and TNF-*α*. This results in T_H_2 polarization [84].

There are a growing number of helminth mediators identified in the secretome that have the potential to be used in new therapeutic strategies against inflammatory diseases. Furthermore, the identification of the mechanisms and pathways these mediators utilize to redirect the immune system might reveal further key mechanisms that have evolved in host-parasite coevolution. Below we provide some examples of immunomodulatory proteins found in the secretome of parasitic nematodes.

## 6. Proteins Found in the Secretome of Parasitic Helminths

The secretome contains functionally diverse classes of molecules that are involved in different vital processes. While some proteins are secreted by exocytosis via the classical pathway using a hydrophobic signal peptide, other alternative pathways include exosomes, lysosomes, and microvesicles. Exosome-like vesicles have been described in the trematodes* Echinostoma caproni* and* F. hepatica*. These extracellular vesicles are internalized by an unspecific endocytic pathway or by specific ligand-receptor recognition mechanisms [85]. Transmembrane flipping and translocation can also result in the release of proteins. Finally, proteins can shed their extracellular domains, while other parts remain inside [86].

Parasitic nematodes secrete a wide range array of proteins and obviously not all of them interact locally and systemically with host immune cells; for example, there are proteolytic enzymes that are secreted to help parasites penetrate the host skin, enable tissue migration, or are involved in feeding. Furthermore, detoxifying enzymes or stress-related proteins are released to assist parasite survival in inflamed tissues. Acetylcholinesterases (AChe) are utilized that potentially interfere with secretion processes of the intestinal mucosa involved in the expulsion of pathogens [87]. Recently, it has been shown that acetylcholine is capable of modulating the activity of macrophages and attenuating local and systemic inflammation [88], making the secretion of AChe by parasites even more intruiging.

Parasitic nematodes include pathogens from plants and animals. Ectoparasitic plant parasites feed on the roots, while endoparasites penetrate the root. The obligate root-knot* Meloidogyne* species have evolved a highly sophisticated relationship with their hosts. Here, secretory proteins play an important role during migration through the roots and the formation and maintenance of proliferating cells [89]. Besides this, just like in animal-infecting parasites, molecules are secreted that are involved in the suppression or evasion of the innate immune system of the host plant. Here, antioxidant proteins coat the surface of the nematode or jasmonic acid-dependent responses are blocked. Furthermore, plant cells are reprogrammed to form multinucleate giant cells as a permanent feeding structure by the induction of nuclear division without cytokinesis [90].

Most secretory proteins of parasitic plant nematodes are produced in the oesophageal, amphidial, and rectal glands, as well as in the hypodermis and intestine [90, 91]. Common secretome components include cell-wall-degrading enzymes and expansins, venom allergen homologues (VAL), SXP/RAL-2 protein, MAP-1, SEC-2, and cuticle collagens [90].

Unlike the previously mentioned nematodes, the pine wood nematode* Bursaphelenchus xylophilus* does not establish permanent feeding sites but kills quickly by feeding on parenchymal cells after migrating through the resin canals of the tree. Following the death of the plant cells, the nematode feeds on fungal growth [79]. Due to this special feeding habit, ES products of the parasite include cell-wall-degrading enzymes like cellulases, pectate lyase, expansin-like, and venom allergen-like proteins. Furthermore, cysteine and aspartic peptidases are two of the most abundantly secreted peptidase groups found in the* B. xylophilus* secretome [79]. These could be beneficial for the parasite in several ways: it either allows the degradation of host molecules for their own nutritional purposes or serves as a defense against host responses [79]. Besides peptidases, 47 peptidase inhibitors were found that could battle against host plant peptidases. Interestingly, expression of host peptidases was significantly increased during* B. xylophilus* infection [79].

In general, animal parasitizing helminths secrete two sets of protease inhibitors that have immunomodulatory properties, cystatins, and serpins. The varying properties of cystatins from parasitic nematodes with respect to their free-living relatives point to the acquisition of anti-inflammatory properties during the coevolution of the parasites and their hosts. Cystatins have been shown to interfere with the host immune cell signaling pathways. They inhibit cysteine proteases such as cathepsins and aspartyl endopeptidase which are important for the processing and presentation of antigens by APCs. Thereby, they inhibit T cell activation. Furthermore, cystatins also prevent T cell proliferation and trigger the decrease in costimulatory molecule expression by APCs [58]. Serpins on the other hand are inhibitors of serine proteases and are able to inhibit neutrophil proteinases and elastase and cathepsin G [92]. The serpin SPN-2 is the most abundant member of secreted proteins from* B. malayi* microfilariae; however, its function is still not clear [93].

To survive within their host, nematodes secrete a battery of diverse antioxidant systems that detoxify oxygen radicals produced by infection-stimulated host phagocytes. These proteins include peroxiredoxin, catalase, glutathione peroxidase, superoxide dismutase, thioredoxin, thiroredoxin peroxidase, and many more [7, 94]. Secretory glutathione S-transferases (GSTs) are thought to participate in the protection of parasite membranes from peroxidation [95]. Interestingly, the secretory GST-1 from* O. volvulus* has prostaglandin D2 activity, thereby contributing to the production of parasite-derived prostanoids [96].

The nematode* Haemonchus contortus* belongs to the order of the Strongylida and can infect both cattle and humans worldwide. This blood feeding nematode elicits haemorrhagic gastritis, anemia, oedema, and associated symptoms by nurturing on capillaries of gastric mucosa [97, 98].* H. contortus* has a large set of secreted peptidases and peptidase inhibitors that function in host penetration, blood feeding, and blood-digestion [97–100].

Similar to the ES products of other parasitic nematodes,* H. contortus* releases substances influencing the host-parasite interaction as well as the host immune response, resulting mostly in a T_H_2 response. ES products also include sugar-binding proteins that act as receptors for glycoprotein ligands. These C-type lectins and galectins mimic host molecules and might facilitate evasion by competing with host lectins for the binding to ligands that are involved in inflammation [58, 98, 101]. Interestingly, galectin-9 from the canine gastrointestinal nematode* Toxascaris leonina* was shown to suppress dextran sulfate sodium-induced intestinal inflammation in mice and elevated levels of IL-10 and TGF-*β* were observed [102].

Other types of molecules that mimic host molecules are IFN-*γ*, TGF-*β*, and the macrophage migration inhibition factors (MIFs) [103]. The cytokine MIF is an early mediator of innate and aquired immune responses and is rapidly upregulated in various inflammatory conditions [104]. Besides having cytokine activity, MIFs also have oxidoreductase and tautomerase activity. The filarial MIF homologue from* B. malayi* promotes alternative activation of macrophages in a T_H_2 environment. This activation can be directly linked to its oxidoreductase activity [105, 106].

ES products from the murine gastrointestinal parasite* Heligmosomoides polygyrus* were shown to have a wide range of immunomodulatory activities including the suppression of airway allergic inflammation [41]. Also, the calcium-binding chaperone calreticulin was shown to induce a T_H_2 response and at the same time interact with the mammalian scavenger receptor type A on DCs [107]. The proteins VAL-1 and AChe-1 are prevalent in L4 and adult ES products. They are considered as antigenic targets, since they induce protective immunity in mice; however, their mode of action is still unknown. While ES products from L4 and adults also seem to have TGF-*β* activity, released molecules from the egg stage appear to be less important in immunomodulation [108]. The Sushi domain protein family and the ShK/SXC domain toxin family are highly prevalent in the L4 secretome [108]. Sushi-like proteins are prevalent in mammals and regulate complement activation. The conserved ShK/SXC domain that shows similarity to cnidarians toxins is also extensively expressed by other nematodes including* T. canis* [108, 109]. Proteins of this family are able to inhibit calcium-dependent lymphocyte activation [110].

The* A. suum* secretome comprises about 750 molecules and contains many peptidases used for penetration and degradation of host tissue and molecules which serve to escape or modulate the host immune response. Secreted peptidases such as astacin, serine-, cysteine-, and metalloproteases ensure migration and feeding of the worm [111]. Besides this, these proteases are involved in the modulation of the host immune response [111–113]. In a murine air pouch model, the* A. suum*-derived protein PAS-1 inhibits the inflammatory leukocyte migration and reduces the synthesis of proinflammatory cytokines. Furthermore, the suppressive effect of PAS-1 in OVA-induced lung allergic inflammation was shown to be attributed to the induction of CD4^+^CD25^+^ T cells and CD8^+^ T cells [114].

The secretome from the canine filarial parasite* Dirofilaria immitis* contains a 15 kDa antigen (DiAg) that can induce antigen-nonspecific IgE production in rats through increased generation of T_H_2-related cytokines. Interestingly, DiAg suppresses the immediate dermal response to allergen-IgE interactions. This supports the IgE blocking hypothesis mentioned above [115].

In* Teladorsagia circumcincta*, an astacin-like metalloprotease and cathepsin F were identified as the most abundant ES products. These proteins are known to digest host proteins; however, the astacin-like metalloprotease additionally stimulates the immune responses during the early phase of the infection [116, 117].

Carbohydrates that are linked to proteins and lipids of nematodes have been shown to have immunogenic and immunomodulatory properties [118]. ES proteins of* A. suum* that are homologous to helminth-secreted peptides with important immunogenic or immunomodulatory roles in host animals are mostly O-linked glycosylated proteins. These glycans are unusual and structurally distinct from host glycans and induce a glycan-dependent cytokine response biased toward Th2 cells [111].

The major antigenic determinant phosphorylcholine (PC) is a small hapten that is often linked to carbohydrate epitopes in gastrointestinal and filarial nematodes [119]. PC-bearing antigens are able to interfere with key proliferative pathways in B and T cells, DC maturation, and mast cell degranulation [120]. The rodent filarial parasite* Acanthocheilonema viteae* secretes the aminopeptidase ES-62, which is the most intensely studied PC-substituted protein. ES-62 exerts its effect on various immune cells, where its anti-inflammatory action depends on the PC-moiety. It has the ability to inhibit B cell, T cell, and mast cell proliferation, promotes the alternative activation of macrophages, and is responsible for the T_H_2 response through inhibition of IL-12p70 production by DCs [121]. In a mouse model for rheumatoid arthritis, ES-62 was able to significantly reduce the severity of developing collagen-induced arthritis and suppress further progression of an already established disease [122] Furthermore, its anti-inflammatory action was also observed in human rheumatoid arthritis-derived synovial tissue cultures [123].

Here we have given a few examples of proteins found in the secretome of parasitic nematodes, some with known functions in immune modulation and some with as-yet hypothetical functions.

Helminth secretomes are a rich source of novel drug and vaccine targets, diagnostic markers, and immunomodulatory proteins. While the analysis of secreted proteins from different life stages of helminths is still quite challenging, numerous secretome analyses of helminths exist by now (Table 2). The combination of the existing data towards a more integrated view of ES products from helminths will be the next logical step. Existing difficulties, such as the lack of genomic sequence information, can be dealt with by using RNA-sequence assembly as reference for the identification of ES products. More challenging, however, are low protein concentrations due to high dilutions of cultivation media, is contamination of normally nonsecreted proteins due to cell lysis and death, or is that most developmental stages cannot be cultivated* in vitro* [117]. Here enrichment methods could be applied that are based on posttranslational modifications of secreted proteins, for example, glycosylation [124].

## 7. Conclusion

Helminthic infections have a large impact on global health and can cause severe forms of helminthiasis. Nevertheless, they have proven to have immunomodulatory and immunoregulatory effects on the host's immune system which can be exploited in the treatment of immune dysregulatory diseases. While helminths have independently evolved various strategies to gain entrance to host tissues and to actively evade or even manipulate the signaling network of the immune system, the host developed strategies to limit pathology by shifting the T_H_2 response towards immunosuppression instead of triggering an inflammatory tissue-damaging response.

A number of promising clinical trials were performed using live worms to treat immune dysregulatory diseases. However, the major research aim is to identify and characterize helminth-derived modulators which can foster anti-inflammatory drug development.

## Figures and Tables

**Table 1:** Overview of the most common human pathogenic helminths.

Organism	Number of people infected (in millions)	Disease pathology
**Nematoda**		

*Ascaris lumbricoides *	807–1121	Impaired digestion, anemia, iron deficiency, poor growth, cough, fever, abdominal discomfort, and passing of worms
*Trichuris trichiura *	795–1050	
*Necator americanus *	740–1300	
*Ancylostoma duodenale *		
*Strongyloides stercoralis *	30–100	

*Wuchereria bancrofti, Brugia malayi, Brugia timori *	120	Chronic lymphoedema, elephantiasis of limbs, and hydrocele

*Onchocerca volvulus *	37	Dermal pathology characterized by pruritus, altered pigmentation, atrophy, and lymphadenitis. Ocular lesions leading to sclerosing keratitis, chorioretinitis, optic nerve disease, and blindness

*Schistosoma mansoni, Schistosoma haematobium, Schistosoma japonicum *	207	Intestinal schistosomiasis characterized by abdominal pain, diarrhoea, and liver enlargement

**Trematoda**		

*Fasciola hepatica, Fasciola gigantica *	2.4–17	Fascioliasis characterized by fever, abdominal pains, and hepatomegaly

*Paragonimus *spp.	23	Chronic cough, chest pain with dyspnoea, and fever

*Opisthorchis viverrini *	10	Palpable liver, obstructive jaundice, cirrhosis, and cholangitis

*Clonorchis sinensis *	15.3	Clonorchiasis characterized by fever and colic pain

**Cestoda**		

*Taenia solium, Taenia saginata*	Not determined	Cysticercosis characterized by infection of the central nervous system
*Echinococcus multilocularis, Echinococcus granulosus *		Alveolar echinococcosis and cystic echinococcosis

Modified according to Perbandt et al. 2014 [7] and CDC report 2013.

**Table 2:** Overview of the proteomic analyses of helminths secretome.

Organism	Order	Principal host	Analyzed stage	Number of identified proteins	Approach used	References
**Nematoda**						

*Ascaris suum *	Ascaridida	Pig	Adults, female	775	Bioinformatics	[111]

*Brugia malayi *	Filariida	Human	Adults, mixed sex	193	Proteomics, bioinformatics	[125]
			Adults, mixed sex	82		[126]
			L3; L3/L4 molting stage; microfilaria; adults, male; adults, female	3 3 36 9 12		[127]

*Dirofilaria immitis *	Filariida	Dog	Adults, mixed sex	110	Proteomics, bioinformatics	[128]

*Ancylostoma caninum *	Rhabditida	Dog	Adults, mixed sex	105	Proteomics, bioinformatics	[129]

*Heligmosomoides polygyrus *	Rhabditida	Rodents	L4; egg released material; adults, mixed sex	214 209 364	Proteomics, bioinformatics	[108]

*Ostertagia ostertagi *	Rhabditida	Cattle	Adults, mixed sex	2	Proteomics, bioinformatics	[130]
			L4 and adults, mixed sex	15	Bioinformatics	[131]

*Haemonchus contortus *	Strongylida	Sheep, goat	Mixed stages; adults, mixed sex	1,457 107	Proteomics	[98]

*Nippostrongylus brasiliensis *	Strongylida	Rat	Adults, mixed sex	3	Proteomics, bioinformatics	[58]

*Strongyloides ratti *	Strongylida	Rat	Adults, mixed sex	2572	Bioinformatics	[132]
			iL3; parasitic female; free-living stage	196 79 35	Proteomics, bioinformatics	[133]

*Teladorsagia circumcincta *	Strongylida	Sheep, goat	Larval stages; L4; adults, mixed sex	18 15 13	Proteomics	[117, 134]

*Trichinella pseudospiralis *	Trichocephalida	Bird	Larval stages	9	Proteomics, bioinformatics	[135]

*Trichinella spiralis *	Trichocephalida	Mammals	L1	13	Proteomics, bioinformatics	[136]

**Trematoda**						

*Dicrocoelium dendriticum *	Plagiorchiida	Ruminants	Adult (exosome-like vesicles);	84	Proteomics, bioinformatics	[137]
			adult (surface);	113		
			adult (ESP);	29		[138]
			tegument	43		

*Fasciola hepatica *	Prosostomata	Cattle, sheep	Larval stages;	22	Proteomics, bioinformatics	[139]
			adults, mixed sex;	26		
			mollusc-dwelling larva;	8		
			adults, mixed sex; dormant larvae	160 26	Proteomics	[140]

*Schistosoma mansoni *	Strigeidida	Human	Cercaria; egg;	72 188	Proteomics, bioinformatics	[141–143]
			cercaria	23		

## Abbreviations

AAM: Alternative activated macrophages
ACE: Acetylcholinesterase
AcES: * Ancyostoma canium* ES products
APC: Antigen-presenting cell
DCs: Dendritic cells
DiAg: * Dirofilaria immitis* antigen
ECM: Extracellular matrix
ES: Excretory/secretory
Fc*ε*RI: High affinity IgE receptors
GST: Glutathione S-transferase
IBD: Inflammatory bowel disease
IFN-*γ*: Interferon-gamma
Ig: Immunoglobulin
IL: Interleukin
LF: Lymphatic filariasis
LPS: Lipopolysaccharide
MGL: Macrophage galactose C-type lectin
MHC: Major histocompatibility complex
MIF: Macrophage migration inhibitory factor
MR: Mannose receptor
NES: * N. brasiliensis* ES products
NK: Natural killer cells
OVA: Ovalbumin
PAMPs: Pathogen-associated molecular patterns
PAS-1: Protein from* A. suum*
PC: Phosphorylcholine
PRRs: Pattern recognition receptors
RELM-*α*: Resistin-like molecule-alpha
SEA: * S. mansoni* egg soluble antigen
TGF-*β*: Transforming growth factor-beta
T_H_: T helper
TLR: Toll-like receptor
TNF-*α*: Tumor necrosis factor-alpha
T_regs_: Regulatory T cells
TSLP: Thymic stromal lymphopoietin
TSO: * Trichuris suis* ova
VAL: Venom allergen/*Ancylostoma* secreted protein-like.
